# Use of a Zenker’s diverticulum overtube for the safe extraction of an unusual gastric foreign body

**DOI:** 10.1055/a-2767-1833

**Published:** 2026-01-28

**Authors:** Jose A. González González, Leticia Santoyo Fexas, Carlos Eugenio González Martínez, Juan Méndez-Benítez, Cesar Augusto Ramos-Matamoros, Andrea Guillén Graf, Aldo Azael Garza

**Affiliations:** 1 103564 Department of Gastroenterology, Hospital Universitario “José Eleuterio González”, Monterrey, Mexico; 2 387240 Instituto Salvadoreño del Seguro Social, Hospital General, San Salvador, El Salvador; 3 609028 Hospital Escuela Universitario, Tegucigalpa, Honduras

Foreign body ingestion is a frequent clinical issue particularly in children and also in adults with intoxication, psychiatric or neurological disorders, or institutionalized patients. Although most objects pass spontaneously, up to 20% require endoscopic removal 1 . Management depends on size and shape: blunt items may seem harmless, but these >2–2.5 cm in diameter or >5–6 cm in length are unlikely to pass the pylorus and often need intervention. The choice of a retrieval device is based on the object’s characteristics and endoscopist preference. A practice trial with a similar item can help. Extraction carries a risk of mucosal injury or cervical esophageal impaction. Overtubes protect the esophageal/pharyngeal mucosa and provide a safe conduit for removal 2 .

We present a 72-year-old man with Parkinson’s disease admitted for vague abdominal discomfort. He was stable, without dysphagia or vomiting. The family later noticed a missing domino tile. Abdominal radiography revealed a rectangular radiopaque object; computed tomography (CT) confirmed a 5 cm plastic foreign body in the gastric body, without perforation or obstruction.

**Fig. 1:**
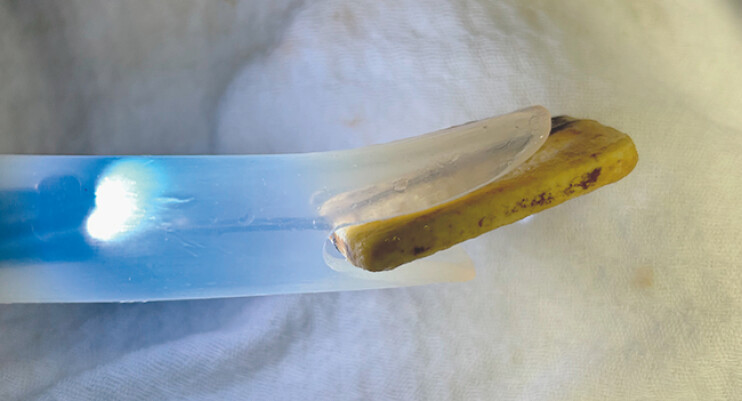
Distal flaps of Zenker’s diverticulum overtube with the ingested foreign body inside.

Given the rigid shape and potential risk of trauma and aspiration, endoscopic removal was attempted using Zenker’s diverticulum overtube ( Video 1 ). Under sedation, the overtube was placed in the upper esophagus. Endoscopy located the domino in the gastric body and fundus. A stone retrieval basket captured the tile, aligned on its long axis, and was retracted toward the scope. The object was guided into the distal flaps of the overtube and then both scope and overtube were withdrawn en-bloc through the esophagus and mouth, achieving safe removal ( Fig. 1 ).

Endoscopic removal of a gastric foreign body using Zenker’s diverticulum overtube to assist in the safe extraction through the upper esophagus.Video 1

This case illustrates the successful endoscopic retrieval of a rigid plastic domino tile using Zenker’s diverticulum overtube. The device provided airway protection and facilitated controlled extraction. To our knowledge, this is the first reported use of this overtube for foreign body removal.

Endoscopy_UCTN_Code_TTT_1AO_2AL
